# Psychometric properties of the Chinese version of the sugar-sweetened beverages media literacy scale for undergraduates

**DOI:** 10.3389/fpubh.2022.1009838

**Published:** 2022-10-14

**Authors:** Chen Long, Myeong Sook Yoon

**Affiliations:** ^1^Health Services Management Department, Guizhou Medical University, Guiyang, China; ^2^Department of Social Welfare, Jeonbuk National University, Jeonju, South Korea

**Keywords:** sugar-sweetened beverages, sugar-sweetened beverages media literacy, undergraduates, psychometric properties, validation

## Abstract

Specific domains of the Sugar-Sweetened Media Literacy Scale (SSM-ML) have been shown to significantly assess sugar-sweetened beverage (SSB) calorie intake in the US population. This study aimed to describe the psychometric properties of the revised Chinese version of the SSB-ML (C-SSB-ML) and evaluate its validity and reliability. Results from 975 undergraduates at two of the largest universities in a province in southwest China showed that Cronbach's alphas for the overall scale, the three dimensions, and two-halves analysis were satisfactory (0.71–0.92). The criterion-related validity of the C-SSB-ML was positively associated with the e Health literacy scale (eHEALS). Confirmatory factor analysis showed that the three-factor model of the C-SSB-ML had adequate fit indices χ^2^ (153) = 4349.93, *p* < 0.001; Comparative fit index (CFI), Tucker-Lewis index (TLI), Incremental fit index (IFI) >0.90; Standardized Root Mean Square Residual (SRMR) <0.07; and Root Mean Square Error of Approximation (RMSEA) <0.08. Our findings provide evidence for a valid and reliable tool that can be used to assess sugar-sweetened media literacy in Chinese undergraduates and will help organizations leverage media literacy in strategy formulation to ensure SSB intake is controlled as much as possible through effective efforts on all fronts.

## Introduction

Consumption of liquid carbohydrates, especially sugar-sweetened beverage (SSB), is not only high but has been increasing globally to varying degrees of significance for decades (1–7). In the United States, per capita consumption of SSB more than doubled from the late 20th century to the early twenty-first century (8), as well as the increase in daily calorie consumption per capita in China of SSB sold by Coca-Cola and PepsiCo by 215 and 147%, respectively, in the decade beginning in 2000 (9). Evidence, including prospective epidemiological studies, suggests that SSB intake is associated with obesity, both in adults and children (1, 10–16). Due to the rapid absorption of liquid carbohydrates, SSB may trigger glucose intolerance and insulin resistance and increase the risk of developing metabolic diseases such as latent autoimmune diabetes in adults, type 2 diabetes mellitus and metabolic syndrome in adults by increasing the dietary glycemic load in the presence of high consumption (1, 11–13, 17–20). SSB intake, even at low calorie levels, promotes higher triglyceride concentrations and is associated with adverse levels of inflammation and serum C-reactive protein, a biomarker of cardiovascular risk, and may lead to an increased risk of cardiovascular morbidity and mortality (13, 16, 20–24). Another issue of concern is the association between the intake of free sugars and dental caries, not only because dental caries is the most prevalent form of NCDs in the world (25–27) but also because the cost of treating dental caries accounts for 5–10% of the health budget of industrialized countries, likely exceeding the entire financial resources spent on children's health in most low-income countries (26–28). Increased SSB intake leads to poor diet quality while consuming more calories (29–31), not only a higher intake of high saturated and high trans fatty acids, but also lack of fiber, vitamins, and essential nutrients (10, 32–36). In addition, high intake of SSB has been observed to be associated with reduced bone mineral compactness and subsequent fractures (37–39).

During the transition from adolescence to adulthood, health-related behavior patterns are being established (40, 41). The transition from home to college may lead to poor choices in undergraduates' diet (40–43). They are in an obesogenic environment, have access to an all-you-can-eat cafeteria, where the food is often high in fat and sugar (17–19). In terms of promoting sound eating habits, undergraduates represent an important subgroup of young adults due to the challenges they face in making healthy choices (20). SSB make up an increasing proportion of calorie intake; during the decade beginning in 2000, SSB sales by Coca-Cola and PepsiCo witnessed an increase in per capita daily calorie consumption in China by 215 and 147%, respectively (21). A national cross-sectional survey in China in 2016 showed that 47% of adults residing in cities consumed more than one serving of SSB per day (22). The problem is that China, sandwiched between the powerful influence of the International Life Sciences Institute (ILSI) and various other SSB companies, remains decades behind in efforts to create a healthier diet for its citizens (23). Media presentations have influenced our perceptions of the foods presented, and the extent to which they now overwhelmingly present and advance foods high in sugar, fat and salt is undeniable (9, 44–48). In terms of promoting sound eating habits, undergraduates represent an important subgroup of young adults due to the challenges they face in making healthy choices (20). Media would now be able to add to this intrinsic inclination as they overwhelmingly present and advance unhealthy food options, with undeniable degrees of sugar, fat, and salt (24–27). Since the 1950s, Western scholars have been concerned about the role of mass media in consumer socialization (28). Media presentations influence our perceptions of the foods showcased and shape our perceptions of what we know, like, and how we should act (29, 30). In 2009, to explore the relationship between smoking and smoking-mediated literacy, antismoking media literacy (AML)—a theoretical framework focusing on skill sets in various domains of media literacy—was developed. Not only did the researchers find that higher smoking media literacy was significantly associated with lower current smoking prevalence, but they also found a strong independent association between higher smoking media literacy and lower susceptibility to future smoking in subsequent analyses (31, 32). Subsequently, based on AML, an ideal internal consistency assessment tool, SSB-ML, was developed (33) and validated among university students in Turkey (34). Result of a UNESCO project survey in China have shown that more than half of college students strongly believe that evaluating the information they find is difficult (49).

Based on the Antismoking Media Literacy (AML) (50, 51), Sugar-Sweetened Media Literacy Scale (SSM-ML), a theoretical framework focusing on skills in various domains of SSB media literacy was developed and validated as an ideal internal consistency assessment tool in American and Turkish populations (52, 53). Based on the gap that the impact of evolving media on eating behaviors has not received sufficient attention, this study aimed to examine the psychometric properties of the C-SSB-ML and to describe the variation in C-SSB-ML scores among undergraduate students with different demographic characteristics in a Chinese setting.

## Materials and methods

### Study design

A non-experimental, cross-sectional study using a survey was conducted the end of September and the beginning of November 2021 with 1,044 students from two of the largest universities located in a province in southwestern China. Participants were selected using incidental and snowball sampling, reaching the target population to the greatest extent possible through paper-based and online questionnaires.

### Translation and adaptation

**Figure 1:**
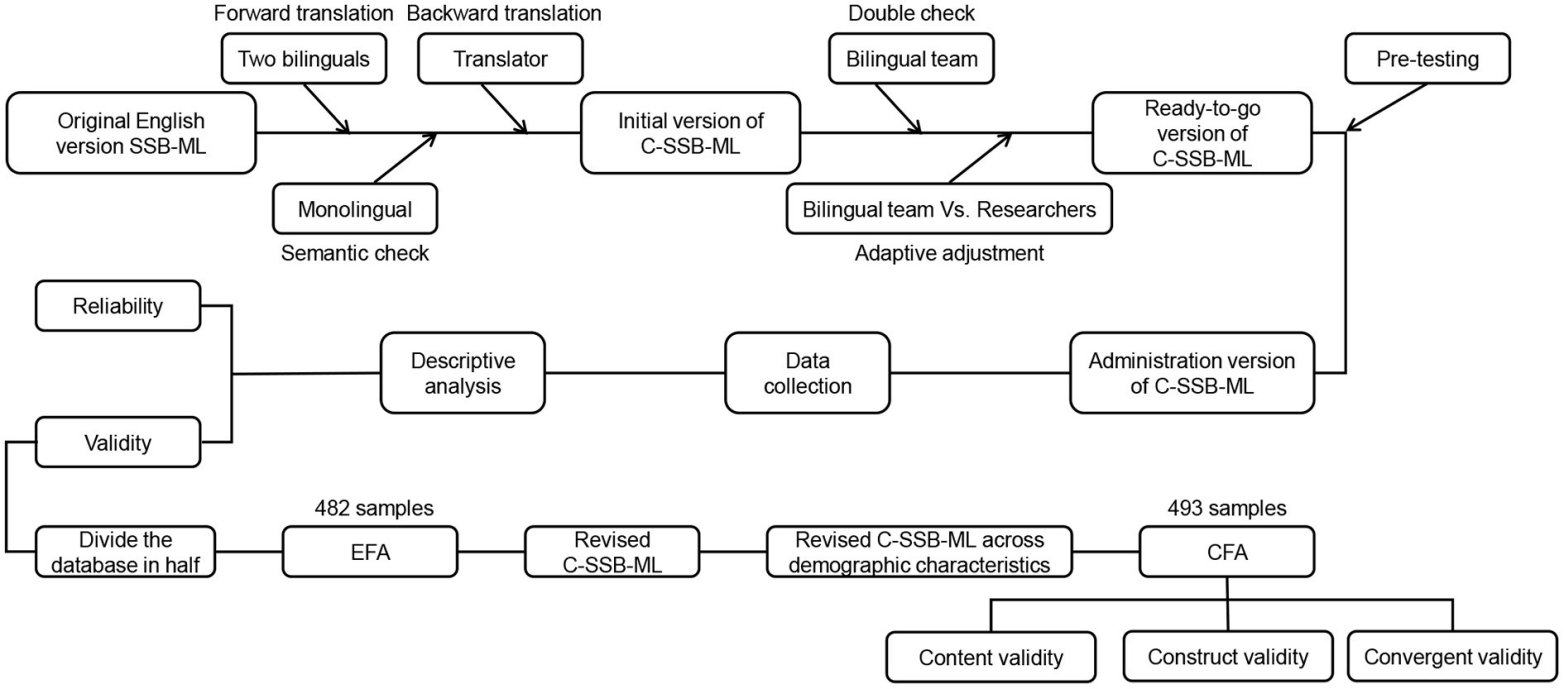
Flowchart of translation and adaptation process and data analysis.

Before beginning the study, e-mail permission was sought from Dr. Yvonnes Chen, one of the main developers of the SSB-ML. In the first step of the translation process, two bilinguals were informed of the basic profile of the study sample population, and the original scale was translated from English to Chinese by these two individuals under the supervision of the study leader. Afterwards, a monolingual who was not good at English was asked to test and comment on the semantic ambiguities of the Chinese version. An English-specialized researcher back-translated the resulting version into English. The bilingual team compares the original scale with the back-translated scale and adjusts it to obtain the C-SSB-ML, Chinese version of SSB-ML. In the process, the words “TV”, “magazine” and “movie” in the original version were adjusted according to the media that Chinese undergraduates encounter in their daily lives and replaced by the words “Douyin (TikTok)”, “short video” and “social media”. The newly developed C-SSB-ML was tested in 20 participants (not included in this study) to check their understanding of the items being used in this study, this was done to assess if all 19 items were easy to provide ratings for and had no ambiguity among Chinese speakers. As the steps in Figure 1.

### Data collection and measures

For the sample size calculation, we carefully considered multiple criteria, firstly in order to have an idea of the problem involved, a careful attempt at a rule of thumb suggests that the sample size should always be more than 10 times the number of free model parameters (54, 55). Based on the six rules of Raykov et al. (56), our model parametric number was determined as follows, with 18 residual variance, 3 covariances between independent variables, and 18 factor loadings connecting the potential variables and their indicators, so our total number of model parameters is 39. Then, the sample size for the first step is determined as 390. Since we want to perform an average and independent database for each of EFA and CFA, our sample size should be at least 780. Also, the criteria for the EFA sample followed Comrey and Lee's suggested size range (57). Considering the response rate and missing values, we tried to reach the maximum number of respondents. Finally, a total of 1,044 participants agreed with the contents. Another inclusion criterion was to have consumed an SSB at least once in the past month and have a clear memory of the type and volume of SSB consumed regularly. Participants had the option of completing the survey through (https://www.wjx.cn/) or through a paper-and-pencil survey provided by their classroom teacher. In the end 975 valid surveys were located, with a response rate of 92.86%. 956 participants chose to complete the survey online, and 19 completed a paper-and-pencil survey, with each one lasting approximately 15–25 minutes.

#### Chinese version of the SSB-ML(C-SSB-ML)

The C-SSB-ML scale was developed to assess individuals' media literacy skills in SSB (52). 19 items are included on the scale. Each item is scored on a seven-point Likert-type scale as “1” for absolutely disagree, “4” for neutral, and “7” for absolutely agree. Functional skills, interactive skills, food selection skills, and critical thinking skills are assessed by this scale (53). The first dimension of the C-SSB-ML, Authors & Audiences (AA), focuses on describing the beverage industry as highly influential and manipulative. The second dimension is Messages and Meanings (MM), which focuses on describing images, symbols, and pictures that evoke an emotional response to achieve a specific marketing purpose. The last dimension involves Representation & Reality (RR), as there is a discrepancy between the real health effects of ingesting either tobacco or SSB and what marketers portray, and this dimension is concerned with that ironic discrepancy (50, 52). The Cronbach's alphas for the entire scale in the previous study were found to be 0.89 and 0.86 (52, 53), with the three sub-dimensions ranging from 0.65 to 0.83 (52).

#### The eHealth literacy scale (eHEALS)

The eHEALS was developed in 2006 (58), measures individuals' multidimensional skills in accessing, understanding, and evaluating health information from electronic media and using this knowledge to solve their health problems. The eHEALS is the first electronic health literacy assessment tool to measure netizens' self-awareness of their ability to seek and apply online health knowledge. There are eight items on the scale, each scored on a five-point Likert-type scale, from “strongly disagree” to “strongly agree”. Sample items include: “I know how to find helpful health resources on the Internet”; “I know how to use the Internet to answer my health questions”; and “I know what health resources are available on the Internet.” The Cronbach's alpha for the overall Chinese version of eHEALS was 0.91 and factor loading coefficients were between 0.69 and 0.87 (59).

#### Socio-demographic questions

This part consisted of five questions about the participant's age, gender, grade, body weight, and height (to calculate the BMI), and mother's education level.

#### Statistical analysis

Confirmatory factor analysis (CFA) was performed with AMOS 24.0, and the rest using SPSS 24.0. Differences in demographic characteristics of C-SSB-ML (overall and the three dimensions) scores and demographic characteristics were analyzed using one-way analyses of variance (ANOVA) and independent sample *t*-tests. Database was divided into two halves, with 482 samples in the first half used to perform explanatory factor analysis (EFA) and 493 samples in the other half used to perform CFA. Substance-factor relationships were determined using EFA. The criteria for factor loading included items having values≥ 0.50 on the primary factor, and no items cross loaded onto other factors. Kaiser-Meyer-Olkin (KMO) statistics and Barlett Sphericity test were used to assess the factorability of the correlation matrix. For factor extraction, the robust maximum likelihood method (RML) with correction for robust mean and variance-scaled was used. An analysis of the CFA was conducted to determine whether the items and dimensions explained the structure of the original scale. To determine whether the items and dimensions explained the structure of the original scale, CFA was performed on a second subsample using the maximum likelihood method (ML). The following criteria are used as cut-off points for ideal fits (60, 61), The goodness-of-fit was assessed by chi-square (χ^2^), chi-square/degrees of freedom (χ^2^/df), the Comparative fit index (CFI), the Tucker–Lewis index (TLI), Incremental fit index (IFI), the Root Mean Square Error of Approximation (RMSEA), and the Standardized Root Mean Square Residual (SRMR). Total score of the C-SSB-ML and discriminant validity among the three dimensions, as well as the convergent validity of eHEALS, were assessed using Pearson correlation analysis. The item-level content validity index (I-CVI) and the average of the I-CVI scores for all items on the scale (S-CVI/Ave) were used to assess the degree of relevance and representation of the elements in the instrument to the goal constructs. C-SSB-ML and three dimensions were tested for internal consistency using Cronbach's alpha coefficients (Figure 1).

### Ethical considerations

This study was approved by the Human Trials Ethics Committee of Guizhou Medical University (dated 22.04.2021 and numbered 2021-LUNSHENDI-150). Respondents were informed of the content of the survey and voluntarily chose to fill out the questionnaire without any compensation, and were informed that they could withdraw at any time without penalty, and that all respondents signed an informed consent form (those who chose to fill out the online questionnaire received an electronic version of the informed consent form).

## Results

### Participant characteristics

The average age of the participants was 19.60 years (SD = 1.44, range = 17–24). Most of the participants were in their first year (74.40%), with the majority being female (61.40%). In terms of their mothers' education, most of participants reported as primary school/below (50.70%), followed by junior high school (27.80%), high school (11%), junior college/above (8.60%), and unaware (1.90%). Using the Working Group on Obesity in China (WGOC) Body Mass Index (BMI) standards (62), the sample was 87.80% underweight or normal (BMI from the lowest to 23.99) and 12.20% overweight or obese (BMI from 24 to 27.99).

### Revised C-SSB-ML across socio-demographic characteristics

First, regarding the mean scores of the dimensions of C-SSB-ML, participants scored the highest on the MM dimension (*M* = 5.25, SD = 1) followed by RR (*M* = 4.88, SD = 1.12) the lowest was AA (*M* = 4.42, SD = 1). The mean score of C-SSB-ML overall was 4.91 (SD = 0.87). Out of a total of 18 items, the highest scoring item was “Two people may see the same short video on Douyin (TikTok) and get very different ideas about it” in the MM dimension (*M* = 5.57, SD = 1.30) and the lowest scoring item was “Certain sugary drink brands are designed to appeal to people like me” in the AA dimension (*M* = 3.94, SD = 1.61). Second, females had significantly higher mean scores than males in both the overall scale and MM dimension, and males had slightly higher mean score than females in the RR dimension but the difference was not statistically significant. Similarly, the three age groups showed statistically significant differences in overall scale and MM dimension score, with the middle age group scoring higher than the other two groups. However, the same pattern was not shown in terms of grade, not only is the difference presented in the scores of the AA dimension, but the highest scores are for 4th year undergraduates. Statistically differences due to BMI were shown in the overall scale, AA dimension and RR dimension, with the most prominent pattern being the coexistence of high BMI and high C-SSB-ML scores (see Table 1).

**Table 1:** Revised C-SSB-ML across socio-demographic characteristics.

	**All participants *n* (%)**	**Overall SSB-ML, mean (*SD*)**	**Authors and audiences, mean (SD)**	**Messages and meanings, mean (SD)**	**Representation and reality, mean (SD)**
**Gender**	
Male	376 (38.6)	4.84 (0.91)	4.44 (1.02)	5.09 (1.02)	4.86 (1.15)
Female	599 (61.4)	4.96 (0.83)	4.41 (0.95)	5.35 (0.97)	4.89 (1.11)
Statistic (*p-*value)		*t* = −2.15, (*p* = 0.03)	*t* = 0.59, (*p* = 0.56)	*t* = −3.99, (*p* = 0.00)	*t* = -0.40, (*p* = 0.69)
**Age**	
≤19	566 (58.1)	4.93 (0.79)	4.38 (0.93)	5.3 (0.91)	4.9 (1.14)
20–21	288 (29.5)	4.95 (0.93)	4.51 (1.02)	5.26 (1.06)	4.91 (1.1)
≥22	121 (12.4)	4.76 (1.01)	4.36 (1)	5 (1.17)	4.7 (1.06)
Statistic (*p-*value)		*F* =3.13, (*p* = 0.04)	*F* = 1.66, (*p* = 0.19)	*F* = 4.71, (*p* = 0.01)	*F* = 2.84, (*p* = 0.06)
**Grade**	
1	725 (74.4)	4.92 (0.84)	4.42 (0.96)	5.26 (0.96)	4.91 (1.14)
2	80 (8.2)	4.82 (0.92)	4.3 (1.03)	5.18 (1.1)	4.81 (1.13)
3	129 (13.2)	4.82 (0.96)	4.32 (1.01)	5.17 (1.13)	4.74 (1.05)
4	41 (4.2)	5.22 (0.9)	4.89 (1.06)	5.5 (1.04)	4.99 (1.02)
Statistic (*p-*value)		*F* = 2.50, (*p* = 0.06)	*F* = 4.06, (*p* =0.01)	*F* = 1.30, (*p* = 0.27)	*F* = 1.00, (*p* = 0.39)
**BMI**
≤23.99	856 (87.8)	4.88 (0.85)	4.37 (0.96)	5.24 (1)	4.85 (1.11)
≥24-27.99	119 (12.2)	5.11 (0.95)	4.8 (1.06)	5.33 (1)	5.09 (1.21)
Statistic (*p-*value)		*t* = −2.70, (*p* =0.01)	*t* = -4.52, (*p* =0.00)	*t* = −0.914, (*p* =0.36)	*t* = -2.21, (*p* =0.03)
**Mother's education level^a^**	
Primary school/below	494 (50.7)	4.86 (0.87)	4.38 (0.99)	5.2 (1)	4.8 (1.11)
Junior high school	271 (27.8)	4.96 (0.84)	4.43 (0.96)	5.29 (0.96)	5.03 (1.08)
High school	107 (11)	5.03 (0.92)	4.53 (0.99)	5.4 (1.07)	4.93 (1.21)
Junior college/ above	84 (8.6)	4.92 (0.87)	4.48 (0.98)	5.24 (1.04)	4.8 (1.11)
Statistic (*p* value)		*F* = 1.58, (*p* = 0.19)	*F* = 0.88, (*p* = 0.45)	*F* = 1.38, (*p* = 0.25)	*F* =2.62, (*p* = 0.05)

a Missing data = 19 (A total of 19 reported they did not know their mother's level of education).

### Psychometric properties

#### Content validity

A panel of nine experts with extensive research experience in the field of health care, comprising three professors/associate professor, one graduate student and one practicing physician, and four PhD candidates. These experts were invited to rate the relevance of the scale's 18 items on a 4-point Likert scale (where 1 = not related, 2 = slightly related, 3 = quite related, and 4 =strongly related). I-CVI is calculated by the percentage of relevance ratings given to items 3 or 4 by content experts, and the average of the proportional correlations of all expert judgments was used to calculate the S-CVI/Ave. The S-CVI/Ave was 0.88 and I-CVI for all questions was equal to or higher than 0.78, and the revised C-SSB-ML had good content validity considering the number of experts (63, 64).

#### Construct validity

According to KMO, sampling adequacy measure was 0.93. The statistical significance of Barlett Sphericity test was χ^2^ (153) = 4349.93 (*p* < 0.001), suggesting that factor analysis can be applied to the data.

In order to further optimize the application model of SSB-ML in the Chinese context, the item with factor loading < 0.5, that is, “sugary drink ads show a healthy lifestyle to make people forget about the health risks, such as weight gain and diabetes” was removed. Subsequently, “wearing a shirt with a sugary drink logo on it makes you a walking advertisement” and “sugary drink in social media link drinking these beverages to things people want, like joy, good looks, and health” were moved to the first dimension. “When designing an advertisement campaign, sugary drink companies think very carefully about the people they want to buy their beverages” and “most social media information shows people drinking sugary drinks make it look more attractive than it really is” were moved to the second dimension. There are two reasons why this alignment was acceptable. First, there was a degree of overlap between the various constructs of media literacy (50, 51). Second, the first two items fit into the original theoretical frameworks of “authors of media messages target specific audiences,” and the latter two also fit into “media messages convey particular values and/or points-of-view” and “producers carefully construct media messages” (50). As Table 2 shows, factor loadings in the AA dimension ranged from 0.51 to 0.68; MM dimension, from 0.56 to 0.80; and RR dimension, from 0.71 to 0.81. In total, 58.94% of the total variance was explained, the first eigenvalue explained 29.40% of the total variance, and the ratio of the first to the second eigenvalue was 1.78, and the ratio of the first to the third eigenvalue reached 2.26, indicating that the scale has the potential to be two-dimensional when used as a survey instrument for Chinese populations.

**Table 2:** Results of the exploratory and confirmatory factor analyses.

**Items**	**EFA**			**CFA**		
	**Factor 1**	**Factor 2**	**Factor 3**	**Factor loading**	**AVE**	**CR**
**Domain 1: “authors and audiences”**	
Grocery store or convenient store deals on sugary drinks, like buy-one-get-one free and other sales, are designed to get people addicted to sugar (1).	0.66			0.55	0.32	0.74
Sugary drink companies are very powerful, even outside of the beverage business (2).	0.66			0.55		
Sugary drink companies only care about making money (3).	0.68			0.57		
Certain sugary drink brands are designed to appeal to people like me (4).	0.68			0.47		
Wearing a shirt with a sugary drink logo on it makes you a walking advertisement (6).	0.51			0.58		
Sugary drink in social media link drinking these beverages to things people want, like joy, good looks, and health (7).	0.54			0.65		
**Domain 2: “messages and meanings”**	
When designing an advertisement campaign, sugary drink companies think very carefully about the people they want to buy their beverages (5)		0.56		0.65	0.53	0.91
Most social media information shows people drinking sugary drinks make it look more attractive than it really is (15).		0.73		0.75		
Two people may see the same short video on Douyin (TikTok) and get very different ideas about it (8).		0.79		0.78		
Different people can see the same sugary drink on Douyin (TikTok) and feel completely different about it (9).		0.75		0.75		
A sugary drink advertisement may catch one person's attention but not even be noticed by another (10).		0.78		0.77		
People are influenced by social media, whether they realize it or not (11).		0.80		0.75		
People are influenced by advertising (12).		0.77		0.68		
When people make short videos, every camera shot is very carefully planned (13).		0.64		0.69		
There are hidden messages in sugary drink advertisements (14).		0.63		0.73		
**Domain 3: “representation and reality”**	
When you see a buy-one-get-one-free or other type of sugary drink sale, it's usually not actually a good deal in the long run (17).			0.81	0.62	0.58	0.80
When you see a sugary drink advertisement, it is very important to think about what was left out of it (18).			0.72	0.79		
Short videos usually leave out a lot of important information (19).			0.71	0.86		
Eigenvalue	7.71	1.78	1.12	
Explained Variance (%)	29.40	16.52	13.02	

The item numbers of the original English version of the scale are indicated in parentheses; *EFA*, exploratory factor analysis; *CFA*, confirmatory factor analysis; AVE, Average variance extracted; *CR*, Composite reliability.

According to the CFA results, loadings of 18 items were medium to high (with values of 0.47–0.87) and the three-factor model for the 18 items exhibited a satisfactory fit as presented in Table 2. Fit indices indicated that three dimensions of C-SSB-ML represented the item responses in Chinese undergraduates with the following values: χ^2^/df = 3.63 (*p* < 0.001), CFI = 0.92, TLI = 0.91, IFI = 0.92, and these values are also considered acceptable (65–68). In addition, this study examined the two model fit indices from an absolute perspective: SRMR and RMSEA. Considering the total sample SRMR value of this study should be close to 0.08 (69, 70), the RMSEA value should be lower than 0.08 for acceptable model fit (60, 69, 70). The results of our study, SRMR of 0.06 and RMSEA of 0.07, are acceptable. Average variance extracted (AVE) and composite reliability (CR) were calculated for each domain, and although the CR values were excellent, one AVE did not reach 0.5, but the results were acceptable because it was higher than 0.3 (71).

#### Convergent validity

The convergent validity of the C-SSB-ML with one external measurement tool. As shown in Table 3, the convergent validity of the C-SSB-ML was also assessed in comparison with the eHEALS score. The eHEALS scores were significantly correlated with the total C-SSB-ML scores, as well as the scores of the three dimensions.

**Table 3:** Correlation between continuous variables of the revised C-SSB-ML and eHEALS.

	**Authors and audiences**	**Messages and meanings**	**Representation and reality**	**SSB-ML**	**eHEALS**
Authors and audiences	1				
Messages and meanings	0.53^***^	1			
Representation and reality	0.54^***^	0.66^***^	1		
SSB-ML	0.80^***^	0.92^***^	0.80^***^	1	
eHEALS	0.19^***^	0.31^***^	0.26^***^	0.31^***^	1

*** *p* < 0.001.

#### Reliability

The Cronbach's alpha was 0.92 for the overall scale. The Cronbach's alpha of the subdimensions were 0.75, 0.92, and 0.82, for AA, MM, and RR, respectively. Based on the two-halves analysis, the Cronbach's alpha for the first and second halves, respectively, were 0.82 and 0.90. The Spearmen Brown coefficient was 0.83, the Guttman split-half coefficient was 0.83, and the correlation coefficient between the two halves was 0.71, which shows the high internal consistency of the scale.

## Discussion

When looking at the C-SSB-ML across demographic characteristics, the first finding was that there was much room for improvement in the C-SSB-ML levels of Chinese undergraduate students. The mean (SD) for SSB-ML overall and across subdomains ranged from 5.83 (0.89) to 6.28 (0.57) for participants of similar age in the study by Chen et al., and were significantly higher than that in our study. However, the similarity between the results of the two studies was that respondents had the highest scores in the MM, followed by the RR, and the lowest in the AA (52).

Our primary finding showed that the relationship between gender and overall score of C-SSB-ML was in line with previous studies stating that females scored higher on the RR, MM, and the scale overall, but only the latter two differences were statistically significant. This suggests that the female participants tended to endorse the pluralistic structure of media literacy as a whole and SSB' critical attitude toward advertising intentions. This is consistent with previous studies in which females scored higher than males in media literacy sets or certain media literacy subdomains (72, 73). Participants with a BMI of 24–27.99 had significantly higher scores on the C-SSB-ML total scale, AA, and RR than those with a BMI less than or equal to 23.99. First, this suggests that higher BMI and higher media literacy scores can co-exist, which is consistent with the findings of a 2020 study (72). Furthermore, combined with the idea that larger female college students are more likely to have critical attitudes toward media images because they are less likely to be identified in them (74), our findings may suggest that such attitudes may be generalized to other media message areas. Second, this result implies that participants with a higher BMI are more sensitive to the marketing motives and techniques of media authors. In addition, fourth-year undergraduates scored significantly higher than freshman to junior undergraduates on all three subdomains and the overall scale, but no difference was significant, implying that fourth-year undergraduates were more willing to take a critical view of marketing techniques in the SSB industry.

Although the EFA results cannot be compared with the original study's findings (52), in a subsequent study, the factor loadings for the three dimensions were reported to be between 0.30 and 0.92, and a total of 49.90% of the variance can be explained by the sum of the three dimensions of the scale (53). Therefore, the current study indicates that the interpretation of factor loadings and scale variances is highly desirable.

In the initial study, Cronbach's alphas >0.65 were reported for the scale (52). In the Turkish version of the study, Cronbach's alphas were reported >0.65 in both the total scale and subdimensions, as well as in the two-halves (53). Our study is slightly higher than these two prior studies in terms of these values, indicating that the C-SSB-ML and previous studies are similar and has strong internal consistency.

In the literature, it is recommended to use CFA to study the structure determined by EFA, and a good fit is indicated if the model fit indicators are >0.90, the χ^2^/df is <5, RMSEA is <0.08, and SRMR is <0.07 (61, 75). The coefficient loadings in CFA in our study also showed satisfactory results between 0.47 and 0.86 (significantly higher than 0.3), and the model fit indicators were consistent with literature criteria.

Regarding convergent validity, the C-SSB-ML was highly correlated with eHEALS scores, a result that further confirmed the validity of the C-SSB-ML due to the highly established study of the applicability of eHEALS in the Chinese sample population.

Although we did our best to make the present study comparable to the initial study (52), there were still some context-based differences, such as we cannot completely exclude the effect due to the small range of BMI in the current sample population. Finally, it is worth noting that Guizhou province is an economically underdeveloped province in southwestern China, and our participants were representative of the undergraduate students in Guizhou. This representation can therefore not be extended to other provinces in China where the situation is quite different. Future investigations should be conducted in more diverse populations, as whether the SSB-ML subdomains are sensitive to different levels of media literacy needs to be further validated; similarly, the association between BMI and caloric intake of SSB in different populations should be further explored. As well as the association of SSB intake with media literacy, should be compared with the association of many other factors considered to be important predictors of this behavior.

## Conclusions

Identifying which part of the media literacy skill set is most closely related to undergraduate students' SSB intake or motivation can help us design specific SSB control interventions. To our knowledge, this is the first study to validate the C-SSB-ML in a Chinese setting. We examined the psychometric properties of the C-SSB-ML and the results of the study point to the C-SSB-ML as a valid and reliable instrument and it may be an important component of SSB control interventions with Chinese undergraduates or young adults, especially because it is feasible and teachable.

## Data availability statement

The original contributions presented in the study are included in the article/Supplementary material, further inquiries can be directed to the corresponding author/s.

## Ethics statement

The studies involving human participants were reviewed and approved by the Human Trials Ethics Committee of Guizhou Medical University (numbered 2021-LUNSHENDI-150). The patients/participants provided their written informed consent to participate in this study.

## Author contributions

CL and MY conceptualized this study, and they were also responsible for the data analysis. MY directed the study. CL was responsible for data gathering and the initial draft of the manuscript. Both authors contributed to this paper and approved the submitted version.

## Conflict of interest

The authors declare that the research was conducted in the absence of any commercial or financial relationships that could be construed as a potential conflict of interest.

## Publisher's note

All claims expressed in this article are solely those of the authors and do not necessarily represent those of their affiliated organizations, or those of the publisher, the editors and the reviewers. Any product that may be evaluated in this article, or claim that may be made by its manufacturer, is not guaranteed or endorsed by the publisher.
